# The hip fracture incidence curve is shifting to the right: A forecast of the age-quake

**DOI:** 10.3109/17453670903278282

**Published:** 2009-10-01

**Authors:** Ulrica Bergström, Håkan Jonsson, Yngve Gustafson, Ulrika Pettersson, Hans Stenlund, Olle Svensson

**Affiliations:** ^1^Division of Surgery and Perioperative Science, Department of OrthopaedicsSweden; ^2^Department of Public Health and Clinical MedicineSweden; ^3^Department of Community Medicine and Rehabilitation Geriatric MedicineSweden; ^4^Department of Pharmacology and Clinical Neurosciences, Clinical Pharmacology, Umeå University, UmeåSweden

## Abstract

**Background** The number of hip fractures has doubled in the last 30–40 years in many countries. Age-adjusted incidence has been reported to be decreasing in Europe and North America, but is there a decreasing trend in all age groups?

**Patients and methods** This population-based study included all hip-fracture patients over 50 years of age (a total of 2,919 individuals, 31% of whom were men) admitted to Umeå University Hospital, Sweden, from 1993 through 2005.

**Results** The incidence of hip fracture declined between the periods 1993–1996 and 2001–2005: from 706 to 625 hip fractures per 10^5^ women and from 390 to 317 hip fractures per 10^5^ men. However, there was a 114% increase in the number of fractures in women aged 90 or older (12 and 25 hip fractures/year, respectively, in the two time periods). For the period 2001–05, women ≥ 90 years of age accounted for almost the same numbers of hip fractures as women aged 75–79 (27 fractures/year). The rate increased during this period, from 2,700 per 10^5^ women to 3,900 per 10^5^ women > 90 years. In men there were declining trends for both relative and absolute numbers.

**Interpretation** Although age-adjusted incidence declined in the population > 50 years of age, absolute fracture rate and incidence increased in the very old. Women over 90 now have the same absolute number of hip fractures every year as women aged 75–79 years. There was a right-shift in hip fracture distribution towards the oldest old, probably due to an increased number of octo/nonagenarians, a new population of particularly frail old people that hardly existed earlier. Better health among septuagenarians may also have delayed the age at which fractures occurred. This changing pattern will strain orthopedic and geriatric resources even more.

## Introduction

Hip fracture is a major and increasing global public health problem. In many countries hospital costs associated with hip fracture already exceed those from all other fractures. In Sweden, it is estimated that the number of people aged 80 and older will double by the year 2050. This increase can be related to the post-World War II baby boom and to the remarkable triumphs of modern medicine and public health.

In recent years a decline in hip fracture incidence has been reported around the world (Jaglal et al 2005, Kannus et al. 2006, Nymark et al. 2006), perhaps because of better general health in the elderly. In epidemiological work, hip fracture patients are implicitly regarded as one homogeneous group, but clinically this population is certainly heterogeneous—perhaps more so than for any other trauma diagnosis. In terms of treatment, nursing and rehabilitation, these patients pose a gigantic challenge. Recent epidemiological studies of secular trends in hip fractures have indicated that, despite the overall decline, the incidence has increased among the oldest old (Hernandez et al. 2006, Lönnroos et al. 2006, Chevalley et al. 2007). Thus, we investigated trends in the incidence of hip fracture, focusing on separate age groups in a population-based material in order to ascertain whether there might be age-specific changes in hip fracture incidence.

## Material and methods

Umeå, Sweden, is situated on the Gulf of Bothnia (64°N). The mean number of inhabitants in the hospital catchment area over the study period 1993–2005 was 137,480 (131,221–142,197). In the population aged 50 and older, the numbers increased from 38,523 to 47,319 during that time. Since the area is served by only one emergency unit and one radiology unit, all fractures requiring treatment in this region are seen at our hospital and therefore registered. All patients who were admitted via the emergency department due to accident/trauma were registered. The unique Swedish personal identification numbers were used. The database was also compared with the hospital registers on an annual basis; thus, all in-hospital fractures were also registered. By cross-checking against the hospital's compulsory E-code registration regarding the reason for hospital admission, any possibility of losing in-patients in the data set was minimized.

The injured person answered a questionnaire about the circumstances of the injury. Data were also collected from ambulance staff, bystanders, and relatives. Information from all the medical records that were available was also included. Treatment at the emergency department involved both in- and outpatients. Validation of the injury database by comparing it to the hospital's radiology archive and surgical database revealed an accuracy of 90% (Bergström et al. 2008). During registration, the database was also continuously controlled by logic validation tests.

The anatomical location of each fracture was recorded in accordance with NOMESCO classification NCECI (NOMESCO, 1997). Both trochanteric and cervical fractures were included. During the period 1993–2005 more than 126,600 injuries, causing 31,173 fractures, were registered; 13,931 of these occurred in patients aged > 50. To eliminate annual variations, we compared the incidence over the period 1993–96 with the incidence over the period 2001–05. The study started in 2004 when we had 3 periods of 4 years each. When the 2005 database was completed, that year was also included in the calculations, making the third period 5 years. This was adjusted for in the calculations and does not affect the results.

### Statistics

The incidence in different age groups was calculated as the number of hip fractures divided by the mean population. The age-adjusted incidence was calculated with a Swedish population at year 2000 as standard population, i.e. when calculating incidence rate for the total population each year, the age distribution 2000 was used for all other years. The incidence rate ratio (IRR) and mean incidence were calculated with 95% CI. The Poisson regression was calculated with overdispersion.

## Results

**Figure 1.**
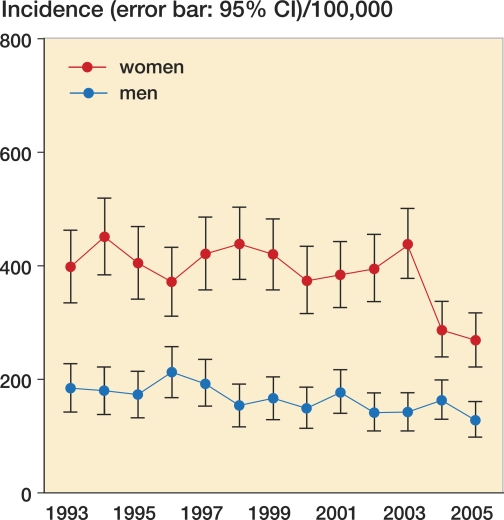
Age-adjusted hip fracture incidence, 1993–2005.

The annual number of hip fractures was fairly constant over time: about 220 cases per year. However, in men and women aged ≥ 50 or older, the age-adjusted incidence showed a declining trend and was 384 per 105 in 2005—compared to an all-time high in 1994 of 592 (IRR = 1.55, 95% CI: 1.37–1.89) (Figure 1). Poisson regression for gender showed an IRR of 1.45 (CI: 1.31–1.60); for age it showed an IRR of 1.79 (CI: 1.75–1.84) and for year it showed an IRR of 0.97 (CI: 0.96–0.99). 69% of the hip fracture patients were women. The most prominent decline in incidence was also seen in women, where the age-adjusted incidence decreased by 35% between 1994 and 2005, but a slight decrease could also be seen in men.

**Figure 2.**
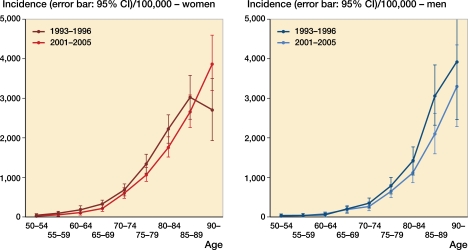
Hip fracture incidences in different age groups, 1993–1996 and 2001–2005.

When comparing the hip fracture incidence from the periods 1993–96 and 2001–05, there was a decrease in all age groups except for women aged ≥ 90 where the incidence increased from 2,700 to 3,860 per 10^5^ (CI: 1.03–2.04), i.e. by 44% (Figure 2).

**Figure 3.**
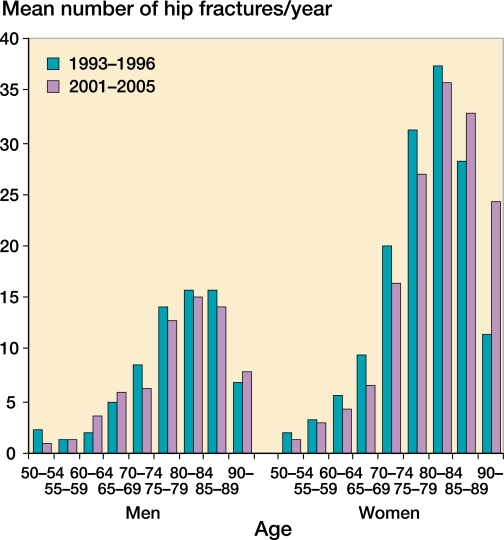
Absolute numbers of hip fractures, 1993–1996 and 2001–2005.

The absolute annual numbers of hip fractures increased in men from 90 years and in women already from 85 years of age. In women aged ≥ 90, the number had more than doubled (12–25 hip fractures/year) (Figure 3).

## Discussion

### Decreasing incidence

In recent studies, the age-adjusted incidence of hip fracture has generally been on the decline, but there are wide variations. In Scandinavia (Kannus et al. 2006) and Canada (Jagal et al. 2005), a decline has been detected from the mid-1990s; but even in as small a region as Scandinavia, the decline in Finland and Denmark (Nymark et al. 2006) seems to have occurred later than in Sweden (Rogmark et al. 1999) and Norway (Giversen et al. 2006). Paspati et al. (1998) detected an 8% annual increase in incidence in Greece between 1977 and 1992. Hernandez et al. (2006) showed that the incidence in Spain was quite stable between 1988 and 2002.

The variations in the onset of the decline in hip fracture incidence may be related to the general health of the population. Cardiovascular morbidity and mortality have decreased during the 1990s. Also, the group consisting of the younger old (60–70 years of age) is increasing drastically due to the baby boom after World War II. When calculating the overall incidence of hip fracture, this relatively healthy group tends to predominate and changes in the oldest old do not stand out.

### Increasing incidence

We found a shift in the overall age-related pattern of hip fracture: an expanding group of elderly is contributing to an increasing number of fractures. Other studies on chronological trends in different age groups have also shown an increase among the oldest old, from the beginning of the twenty-first century. Studies from Spain (Hernadez et al. 2006) and Switzerland (Chevalley et al. 2007) have highlighted this rise, and a recent Finnish study (Lönnroos et al. 2006) showed a 1.7-fold increase in hip fracture incidence in the oldest old (> 75 years).

**Figure 4.**
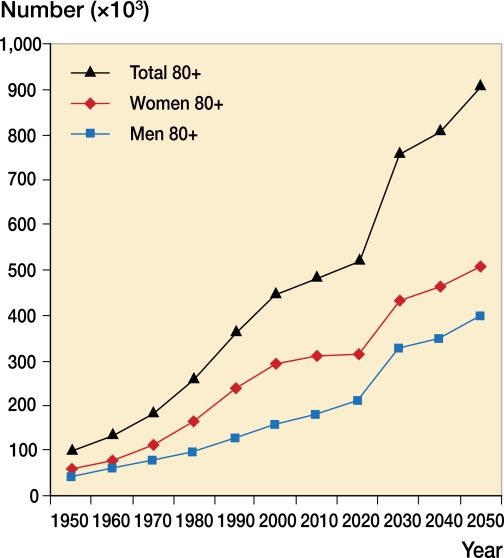
Expected changes in the Swedish population > 80 years of age (SCB).

Postponement of the date of fracture and adding more “healthy” years to life is in itself a good thing. But the increasing incidence of fracture in the oldest old represents a frightening forecast of the scenario that is bound to occur when the age-quake of World War II baby boomers will occur. In Sweden, the number of inhabitants aged 80 and older will have doubled by the year 2050 (Figure 4), and this phenomenon will also be seen globally.

### The fall and rise in fracture incidence

**Figure 5.**
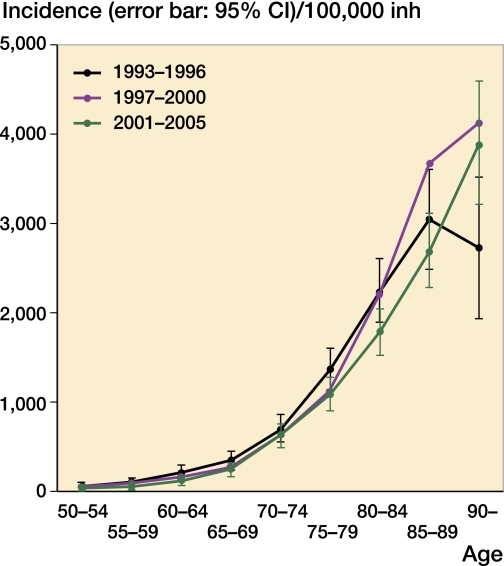
Hip fracture incidence in women, 1993–2005.

In the cohort of individuals aged 90 years and older, there was an increase in hip fracture incidence and prevalence. When analyzing this cohort further, we found that this change appeared to have a progressive course of events. Looking at the incidence for 1997–2000, the curve showed a moderate increase in incidence in this age group compared to 1993–96. The the curve for 2001–2005 indicates a more pronounced increase (Figure 5). Perhaps the 100+ cohort would now show a similar drop in fractures as the 90+ cohort had 10 years ago. This is perhaps because a larger population of the oldest old was less mobile in 1993–1996 compared to the same age cohort 10 years later. A bedridden person in a nursing-home has a low risk of falling, while a mobile 90-year-old (or older) in a group dwelling for people with dementia has the highest risk.

### The age-quake

During the period 1993–2005, the increase in the number of nonagenarians was 77% (534 to 947) in the Umeå region. Assuming a similar trend all over the Western world, widespread longevity will be one of the major challenges facing future healthcare. Due to better treatment of cardiovascular disease and cancer, for example (Picirillo et al. 2008), an increasing proportion of people may justifiably expect to become very old. Instead of succumbing to myocardial infarction or cancer, they survive to be afflicted by other degenerative processes. Several studies have reported an increasing survival rate after stroke (Hallström et al. 2008) and preliminary results from the catchment area of Umeå University Hospital indicate an increased age-adjusted prevalence of dementia (Gustafson et al. unpublished). This is probably also partly due to improved care and thus prolonged survival of dementia patients, but also due to more people surviving with vascular risk factors associated with the development of dementia. In our catchment area, 30% of people aged ≥ 90 years have suffered a stroke and about 50% are afflicted with some kind of dementia (Von Heideken et al. 2006). Very old people are being prescribed an increasing number of drugs that are associated with an increased risk of falling, such as antidepressants and benzodiazepines (Lövheim et al. 2007). This means that a large proportion are living with diseases and/or drug treatments that increase the risk of hip fracture. We have previously reported a more than doubled proportion of patients afflicted by stroke (Ramnemark et al. 2000) and a 50% increase in subjects suffering from dementia among hip-fracture patients during the last x decades (Gustafson et al 1991). Stroke and dementia both increase the risk of hip fracture two- to fourfold (Ramnemark et al. 1998), as well as the risk of postoperative complications. Outcome for the oldest fracture patients differs from that for the younger ones, as they stay longer in hospital, are less likely to recover prefracture ambulatory ability, and carry a higher risk of heart failure and a twofold higher risk of pulmonary infection (Roche et al. 2005).

Cardiovascular health has improved in the elderly. Now it is time to concentrate upon improving skeletal health in the very old. In the age group ≥ 80 years, osteoporosis is by definition a very common condition; almost 50% have a BMD of < 2.5 SD. Low BMD is not a problem in itself, but it comes with the risk of a future fracture, and more subjects who are mobile are at a higher risk of having a fall followed by a fracture. The role of bone resorption inhibitors in the elderly requires further investigation. Most studies on medicinal drugs are done on a healthy “young” population. Limited resorption of the active substance and the gastrointestinal inconveniences of bisphosphonates have contributed to their very limited use in the oldest old. New bisphosphonate formulae suitable for monthly or annual infusion may be more feasible for this category of patients.

### Conclusion

This study confirms reports of a reduced incidence of hip fracture in the younger elderly population, but we also found an increased incidence in the very old population; the hip fracture curve is shifting to the right. Considering expected demographic changes, hip fracture in the very old will be a challenge for healthcare systems. Coping strategies include optimal treatment of osteoporosis, effective prevention of falls, advanced anesthesiological and surgical techniques, and active postoperative rehabilitation. The outcome of hip surgery is less favorable in this age group. Thus, along with fracture prevention we need to reconsider the organization of surgery, nursing, and rehabilitation of the oldest old.
